# Theoretical Analysis of Light-Actuated Self-Sliding Mass on a Circular Track Facilitated by a Liquid Crystal Elastomer Fiber

**DOI:** 10.3390/polym16121696

**Published:** 2024-06-14

**Authors:** Lu Wei, Junjie Hu, Jiale Wang, Haiyang Wu, Kai Li

**Affiliations:** School of Civil Engineering, Anhui Jianzhu University, Hefei 230601, China; weilu@ahjzu.edu.cn (L.W.); 13966837296@163.com (J.H.); wjl1230504@163.com (J.W.); hywu@stu.ahjzu.edu.cn (H.W.)

**Keywords:** self-sliding, liquid crystal elastomer, light-actuated, sliding mass, curved track

## Abstract

Self-vibrating systems obtaining energy from their surroundings to sustain motion can offer great potential in micro-robots, biomedicine, radar systems, and amusement equipment owing to their adaptability, efficiency, and sustainability. However, there is a growing need for simpler, faster-responding, and easier-to-control systems. In the study, we theoretically present an advanced light-actuated liquid crystal elastomer (LCE) fiber–mass system which can initiate self-sliding motion along a rigid circular track under constant light exposure. Based on an LCE dynamic model and the theorem of angular momentum, the equations for dynamic control of the system are deduced to investigate the dynamic behavior of self-sliding. Numerical analyses show that the theoretical LCE fiber–mass system operates in two distinct states: a static state and a self-sliding state. The impact of various dimensionless variables on the self-sliding amplitude and frequency is further investigated, specifically considering variables like light intensity, initial tangential velocity, the angle of the non-illuminated zone, and the inherent properties of the LCE material. For every increment of π/180 in the amplitude, the elastic coefficient increases by 0.25% and the angle of the non-illuminated zone by 1.63%, while the light intensity contributes to a 20.88% increase. Our findings reveal that, under constant light exposure, the mass element exhibits a robust self-sliding response, indicating its potential for use in energy harvesting and other applications that require sustained periodic motion. Additionally, this system can be extended to other non-circular curved tracks, highlighting its adaptability and versatility.

## 1. Introduction

Self-vibration exists widely in natural phenomena and engineering applications [1,2,3]. It is a phenomenon wherein the system changes periodically under consistent external stimuli [4,5,6], equivalent to stimulation, controlling the phase of the movement. A self-vibrating system typically comprises vibrating elements, reliable energy sources and mechanisms for feedback control [7,8]. In particular, self-vibration is a steady-state cycle movement maintained by the system itself, which means that it can be sustained without the external force of periodic change [9]. This is why it is fundamentally different from forced vibration; hence, self-actuated vibration merits in-depth investigation [10,11,12]. Self-actuated vibration has varied positive aspects, namely, self-actuated vibration is capable of constantly maintaining periodic motion without periodic external stimuli and additional manual control components [13,14], which greatly reduces the requirements for system motion regulation, eliminating the necessity of designing intricate control systems [15,16,17]. Furthermore, this characteristic enables the control system to directly draw energy from a consistent external source to sustain periodic motion [3,4,5]. The self-vibration phenomenon exists in many fields, for instance, non-linear friction inspirational self-vibration [2], steering wheel vibration [11], rotor vortex [18], fluid incentive tremor [13], fluid electromagnetic vibration [19], chemical reaction system vibration [9], and similar instances.

In recent years, significant advancements have been achieved in the research on self-vibration systems, among which, active material-based self-vibration systems have attracted widespread research interest [20,21,22,23,24]. Active materials can respond to various external stimuli to undergo deformation and movement, such as light [2,3], heat [1], electricity [6], magnetism [19,23], etc. Light stands out among the various stimuli due to its distinct advantages, including environmental friendliness [25,26,27], precise controllability, contact-free nature, repeatability, and multifunctionality. In addition, with the intention of upsetting the system’s equilibrium, advanced feedback systems have been established for energy compensation [6,9]. This disturbance prompts a steady and enduring response from the active material, subsequently leading to self-vibration. Examples of such systems include the self-adjusting shading device [28], the integration of large-scale deformation with chemical reactions [9], the connection of fluid evaporation and structural deformation [29], and gradients in surface tension due to photothermal heating [30,31]. The active materials that have the potential to produce self-actuated vibration phenomena include, but are not restricted to, hydrophilic polymer gel [32,33], ionic polymer gel [34], and liquid crystal elastomers (LCEs) [35,36,37].

Among these numerous active materials, LCEs are a Liquid Crystal Polymer material synthesized through the cross-linking of liquid crystal monomer molecules. When synthesizing liquid crystal elastomers, different intermediate molecules can be introduced to create different types of LCEs, providing them with the ability to react to multiple external triggers, including light [38], heat [39,40], electricity [41], magnetism [33], and so forth. LCE materials generally have numerous advantages, such as a rapid deformation response, recoverable deformation, and no noise [42,43,44]. Compared with other active materials categories, these materials have distinctive advantages, such as wireless non-contact driving, a lightweight structural design, and a reduced environmental impact [45,46]. Considering the advantageous characteristics of light, there is a wide range of self-vibrating systems enabled by light-actuated liquid crystal elastomers (LCEs), encompassing actions like bending [47,48], synchronization [49], rolling [50,51], shuttling [52], jumping [31], flying [53], floating [16], swimming [22], spinning [54], chaos [55,56], and various other self-vibration mechanisms. Light-responsive LCEs exhibit extensive applicability and broad prospects in the fields of micro robots [57,58,59], biomimetic soft robots [60,61,62,63], biomedicine [64], and energy harvesting [65], due to their reversible contraction [66] and relaxation properties [67,68] under light stimuli.

There have been many studies on self-vibration based on liquid crystal elastomers, especially the self-vibration mode. However, the diversity of self-vibration modes is not sufficient, and the construction of self-vibration systems is not systematic, which limits the application of self-vibration phenomena in many fields, such as energy harvesting, soft robots, medical equipment, amusement equipment and micro nano devices. In summary, it is imperative to fabricate additional LCE self-vibration structures which exhibit simplicity, controllability, and integrability. In light of this, the paper creatively proposes the LCE fiber–sliding mass system, which consists of a light-actuated LCE fiber, a sliding mass, and a rigid circular track, and investigates several critical aspects, including the conditions for obtaining self-sliding modes, the dynamic mechanisms underlying self-sliding, and the influence of system dimensionless variables on self-sliding modes, amplitudes, and periods. Compared with previous self-oscillating systems, the proposed system features a simplified structure, rapid response, high controllability, and multifunctionality. Importantly, the system’s adaptability extends beyond circular tracks, accommodating a range of non-circular curved paths as well. The objective is to develop a novel light-actuated self-sliding system based on active materials.

The structure of this paper is outlined below. Firstly, based on the available dynamic behavior model of LCE materials and the theorem of angular momentum, the equations for dynamic control of the sliding mass enabled by LCE fibers are deduced in Section 2. Next, the two unique motion states of the LCE fiber–sliding mass system, namely, the static state and self-sliding state, are presented, and a detailed explanation of the operational principle of self-sliding is presented in Section 3. Then, a quantitative analysis is performed to explore the impact of diverse dimensionless variables on both the amplitude and frequency characteristics of self-sliding of the system, as explained in Section 4. Finally, this study’s critical conclusions are precisely abstracted in Section 5.

## 2. Theoretical Model and Formulation

This section begins with a description of a light-actuated self-sliding system, which includes an LCE fiber, a sliding mass, and a rigid circular track. Subsequently, we derive the equations used for dynamic control of the sliding mass enabled by LCE fibers based on the dynamic model of LCE, the theorem of angular momentum, and vibrating theory. Finally, the nondimensionalization and the numerical solution method of the dynamic control equation are introduced.

### 2.1. Dynamics of Self-Sliding System

**Figure 1:**
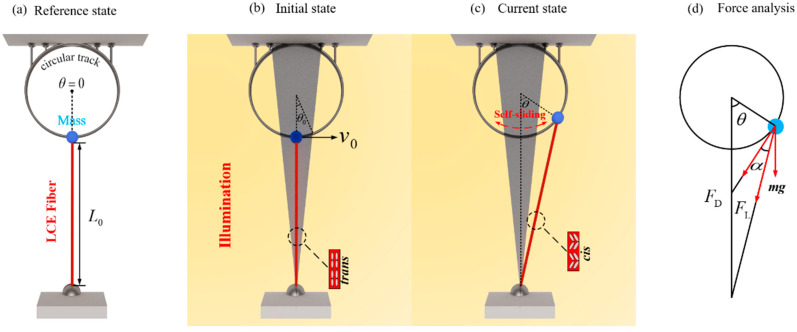
Diagram of the side view of a self-sliding system comprised of a light-actuated LCE fiber, a sliding mass, and a rigid circular track: (**a**) reference state; (**b**) initial state; (**c**) current state; and (**d**) force analysis. In the self-sliding model, the sliding mass with the LCE fiber can slide continuously and periodically along the rigid circular track under sustained illumination.

Figure 1 and Video S1 schematically present a dynamic simulation of a self-sliding actuated system via light, which is capable of sliding continuously and stably under designated initial tangential speed and illumination conditions. The self-sliding system comprises an azobenzene-based LCE fiber, a sliding mass, and a rigid circular track. The photosensitive molecules in LCE fibers, such as azobenzene molecules, are aligned along the fiber axis. The lower end of the LCE fiber is fastened to a horizontal fixed support, while the upper end is linked to a sliding mass with a weight of m. The sliding mass is mounted on the rigid circular track and can slide on it, while the upper end of the track is fixed to a horizontal rigid base. In addition, the weight of the LCE fiber is much lower than the weight of the sliding ball, so it is neglected in this study. In the reference state, the radius of the rigid circular track is denoted as r and the LCE fiber is stress-free with an original length of $L_{0}$, as shown in Figure 1a. We designate the starting position of the sliding mass as the center point of the polar coordinate system, establishing the polar axis, which extends in a radial direction from the core of the circular track. Since the sliding mass is set on the rigid circular track, it can only slide along the tangential direction of the track, with an angular displacement of θ.

As depicted in Figure 1b,c, the bright section denotes the illuminated region, while the gray triangular section denotes the shadowed region, i.e., the non-illuminated region, with the angle from the origin to the first intersection point between the right side of the shade and the circular track being denoted as $\theta_{0}$. Due to the initial tangential velocity in the tangential direction, the sliding mass continues its right counterclockwise motion until it enters the illuminated region. Within this region, the azobenzene molecules in the LCE fibers can absorb light energy and isomerize from straight trans to bent cis, so the LCE fibers contract. As the LCE fiber contracts and extends within the illuminated region, the system’s elastic potential energy peaks when the sliding mass reaches maximum angular displacement. Subsequently, propelled by the tensile force in the LCE fiber, the sliding mass slides in the reverse direction. Upon entering the non-illuminated region, the light-actuated shrinkage of the LCE fiber is restored, resulting in a decrease in its tension until it reaches the lighted region on the opposite side. In this way, it stores potential energy from its elastic deformation and subsequently repeats the entire process. By appropriately selecting system variables and initial conditions, the light-actuated sliding mass structure can sustain consistent and steady self-sliding motion.

The sliding mass bears a tensile force $F_{L}$ from the LCE fiber; the damping force $F_{D}$, and its gravity mg, as depicted in Figure 1d. Along the tangential direction, the dynamic control equation of the sliding mass can be expressed as follows [69]:

(1) $$mr^{2}\ddot{\theta }=-mgsin\theta \cdot r-F_{L}cos\alpha \cdot r-F_{D}\cdot r$$

where $\ddot{\theta }$ represents the acceleration of the sliding mass; $F_{L}$ denotes the tensile force of the LCE fiber; $F_{D}$ refers to the damping force; θ is an angular displacement, measured relative to the vertical line, with a counterclockwise direction designated as positive; α is the angle between the force $F_{D}$ and the force $F_{D}$; g is the gravitational acceleration.

According to the geometric relations in Figure 1d, $cos\alpha =\frac{\left(L_{0}+r\right)sin\theta }{\sqrt{r^{2}+{\left(L_{0}+r\right)}^{2}-2r\left(L_{0}+r\right)cos\theta }}$, where $L_{0}$ is the initial length of the LCE fiber in a stress-free condition and r is the radius of the rigid circular track.

The tension of the LCE fiber is assumed to be proportional to elastic strain, and can be expressed as [70]:

(2) $$F_{L}=KL_{0}\varepsilon_{e}\left(t\right)$$

where K represents the elastic coefficient of LCE; $\varepsilon_{e}\left(t\right)$ denotes the elastic strain in the LCE fiber. For simplicity, the elastic strain $\varepsilon_{e}\left(t\right)$ under small deformation can be assumed to be a linear combination of the total strain $\varepsilon_{\mathrm{tot}}\left(t\right)$ and the light-actuated contraction strain $\varepsilon_{L}\left(t\right)$, i.e., $\varepsilon_{\mathrm{tot}}\left(t\right)=\varepsilon_{e}\left(t\right)+\varepsilon_{L}\left(t\right)$. Therefore, the tension of LCE fiber in Equation (2) can be rewritten as:

(3) $$F_{L}=KL_{0}\left(\varepsilon_{\mathrm{tot}}\left(t\right)-\varepsilon_{L}\left(t\right)\right)$$

For simplicity, the total strain $\varepsilon_{\mathrm{tot}}\left(t\right)$ is defined as $\varepsilon_{\mathrm{tot}}\left(t\right)=\frac{L-L_{0}}{L_{0}}.$ Thus, the tension of $F_{L}$ in Equation (3) can be rewritten as:

(4) $$F_{L}=K(L-L_{0}\left(1+\varepsilon_{L}\left(t\right)\right))$$

where L is the length of the LCE fiber under stress, which can be expressed as $\sqrt{r^{2}+{\left(L_{0}+r\right)}^{2}-2r\left(L_{0}+r\right)cos\theta }$ based on the cosine theorem of a triangle.

For simplicity, when the velocity is low, the damping force is assumed to be approximately a quadratic function, which is always opposite to the direction of motion:

(5) $$F_{D}=\beta_{1}r\dot{\theta }+\beta_{2}r^{2}{\dot{\theta }}^{2}$$

where $\beta_{1}$ and $\beta_{2}$ represent the linear and quadratic damping coefficients, and $\dot{\theta }$ refers to the angular speed of the system.

By inserting Equations (4) and (5) into Equation (1), considering the counterclockwise and clockwise motion directions, we can obtain:

(6) $$mr\ddot{\theta }=-sgn\left(\theta \right)mgsin\theta -sgn\left(\theta \right)Kr\left(1-\frac{\frac{L_{0}}{r}\left(1+\varepsilon_{L}\left(t\right)\right)}{\sqrt{1+{\left(1+\frac{L_{0}}{r}\right)}^{2}-2\left(1+\frac{L_{0}}{r}\right)cos\theta }}\right)(1+\frac{L_{0}}{r})\sin\theta -\beta_{1}r\dot{\theta }-\beta_{2}r^{2}\dot{\theta }\left|\dot{\theta }\right|$$

### 2.2. Dynamic LCE Model

This part primarily illustrates the dynamic behavior of the light-actuated contraction in LCE fibers. For simplicity, the contraction of LCE fibers under small deformation is assumed to be a linearly related to the number fraction φ(t) cis-isomer in LCE fibers, i.e.,

(7) $$\varepsilon_{L}\left(t\right)=-C_{0}\varphi \left(t\right)$$

where $C_{0}$ denotes the contraction coefficient of LCE fibers.

The light-actuated strain of LCE fibers during contraction is dependent on the cis-isomer number fraction *φ*(*t*) in the LCE. According to the research, the cis-isomer number fraction can be manipulated through exposure to UV light or laser irradiation at wavelengths specifically below 400 nm [71]. However, the precise wavelength within this range significantly impacts the efficiency and dynamics of the isomerization process, with 365 nm being a common choice for azobenzene-based systems due to its effectiveness in triggering the trans-to-cis transition [72]. The cis-isomer number fraction depends on thermally excited *trans*-to-*cis* isomerization, thermally driven relaxation from *cis* to *trans*, and light-driven *trans*-to-*cis* isomerization. Typically, thermally excited *trans*-to-*cis* isomerization can be neglected relative to light-powered *trans*-to-*cis* isomerization. Therefore, the number fraction of the cis-isomer in the LCE is governed by the following equation [73,74,75]:

(8) $$\frac{\partial \varphi }{\partial t}=\eta_{0}I\left(1-\varphi \right)-\frac{\varphi }{T_{0}}$$

where $\eta_{0}$ refers to the constant for light absorption, $T_{0}$ denotes the duration of thermally induced relaxation process from *cis* to *trans*, and I represents the intensity of light. By solving Equation (8), the number fraction of cis-isomer can be deduced as:

(9) $$\varphi \left(t\right)=\frac{\eta_{0}T_{0}I}{\eta_{0}T_{0}I+1}+\left(\varphi_{0}-\frac{\eta_{0}T_{0}I}{\eta_{0}T_{0}I+1}\right)exp\left[-\frac{t}{T_{0}}\left(\eta_{0}T_{0}I+1\right)\right]$$

where $\varphi_{0}$ is the initial *cis* number fraction at t=0.

In the region that is illuminated, the initial number fraction is $\varphi_{0}=0$. Then, Equation (9) can be simplified as:

(10) $$\varphi \left(t\right)=\frac{\eta_{0}T_{0}I}{\eta_{0}T_{0}I+1}\left\{1-exp\left[-\frac{t}{T_{0}}\left(1+\eta_{0}T_{0}I\right)\right]\right\}$$

In the region that is non-illuminated, when the light intensity is set to I=0, we can derive the following:

(11) $$\varphi \left(t\right)=\varphi_{0}exp\left(-\frac{t}{T_{0}}\right)$$

where $\varphi_{0}$ can be designated as the peak value of φ in Equation (9), i.e., $\varphi_{0}=\frac{\eta_{0}T_{0}I}{\eta_{0}T_{0}I+1}$. Then, Equation (11) can be simplified as:

(12) $$\varphi \left(t\right)=\frac{\eta_{0}T_{0}I}{\eta_{0}T_{0}I+1}exp\left(-\frac{t}{T_{0}}\right)$$

### 2.3. Nondimensionalization

To simplify calculations, enhance solution efficiency, and improve generality, the following quantities are subjected to nondimensionalization: $\bar{t}=t/T_{0}$, $\bar{\dot{\theta }}=\dot{\theta }T_{0}$, $\bar{\ddot{\theta }}=\ddot{\theta }T_{0}^{2}$, $\bar{g}=gT_{0}^{2}/r$, $\bar{K}=KT_{0}^{2}/m$, $\bar{I}=\eta_{0}T_{0}I$, $\bar{\beta_{1}}=\beta_{1}T_{0}/m$, $\bar{\beta_{2}}$=$\beta_{2}r/m$ and $\bar{\varphi }=\frac{\varphi \left(\eta_{0}T_{0}I+1\right)}{\eta_{0}T_{0}I}$. Thus, Equation (6) can be written in a dimensionless form as:

(13) $$\bar{\ddot{\theta }}=-sgn\left(\theta \right)\bar{g}sin\theta -sgn\left(\theta \right)\bar{K}\left(1-\frac{\frac{L_{0}}{r}\left(1+\varepsilon_{L}\left(t\right)\right)}{\sqrt{1+{\left(1+\frac{L_{0}}{r}\right)}^{2}-2\left(1+\frac{L_{0}}{r}\right)cos\theta }}\right)(1+\frac{L_{0}}{r})\sin\theta -\bar{\beta_{1}}\bar{\dot{\theta }}-\bar{\beta_{2}}\bar{\dot{\theta }}\left|\bar{\dot{\theta }}\right|$$

In the region that is illuminated, Equation (11) can be expressed in another way, as:

(14) $$\bar{\varphi }\left(t\right)=1-\exp\left[-\bar{t}\left(\bar{I}+1\right)\right]$$

In the region that is non-illuminated, Equation (12) can be rewritten as:

(15) $$\bar{\varphi }\left(t\right)=\exp(-\bar{t})$$

Meanwhile, the tension of the LCE fiber in Equation (4) and the damping force in Equation (5) can be written in a dimensionless form as:

(16) $$\bar{F_{L}}=\bar{K}\left(1-\frac{\frac{L_{0}}{r}\left(1+\varepsilon_{L}\left(t\right)\right)}{\sqrt{1+{\left(1+\frac{L_{0}}{r}\right)}^{2}-2\left(1+\frac{L_{0}}{r}\right)cos\theta }}\right)(1+\frac{L_{0}}{r})\sin\theta$$

(17) $$\bar{F_{D}}=\bar{\beta_{1}}\bar{\dot{\theta }}+\bar{\beta_{2}}\bar{\dot{\theta }}\left|\bar{\dot{\theta }}\right|$$

The dynamics of the light-actuated sliding mass system are governed by Equations (13)–(15), where the time-dependent number fraction of cis-isomer is intricately linked to the position of the sliding mass. Making use of dimensionless and given variables, including $\bar{I}$, $C_{0}$, $\bar{g}$, $\bar{K}$, $\bar{\beta_{1}}$, $\bar{\beta_{2}}$, and $\theta_{0}$, we can derive the temporal evolution of the light-actuated contraction strain and the position of the sliding mass. Equations (13)–(15) are evidently nonlinear differential equations, and it is challenging to acquire the analytical calculation. In the study, the standard fourth-stage Runge–Kutta method and MATLAB R2021a software are applied to numerically calculate the steady-state responses, including the light-actuated contraction strain of the LCE fiber, and the angle and angular velocity of the sliding mass at any time. Moreover, we can further obtain the tension of the LCE fiber from Equation (16) and the damping force from Equation (17).

## 3. Two Motion States and Mechanism of Self-Sliding

Rooted in the control equations deduced in Section 2, in this section we discuss the dynamics of the light-actuated sliding mass system under a state of steady illumination. Two typical dynamic modes of self-sliding are first presented, which are categorized as the static state and self-sliding state. Subsequently, we describe the corresponding mechanism of self-sliding.

### 3.1. Two Motion States

To further investigate the self-sliding motion behavior of the LCE fiber–sliding mass system, it is first necessary to determine the typical values of the parameters in the dimensionless equations in Section 2. Based on previously studied outcomes and experimental results [76,77,78], the empirical values of the parameters required in a sliding mass system are collected and presented in Table 1. Using the variable data from Table 1, the corresponding dimensionless variable values can be derived and are listed in Table 2.

**Figure 2:**
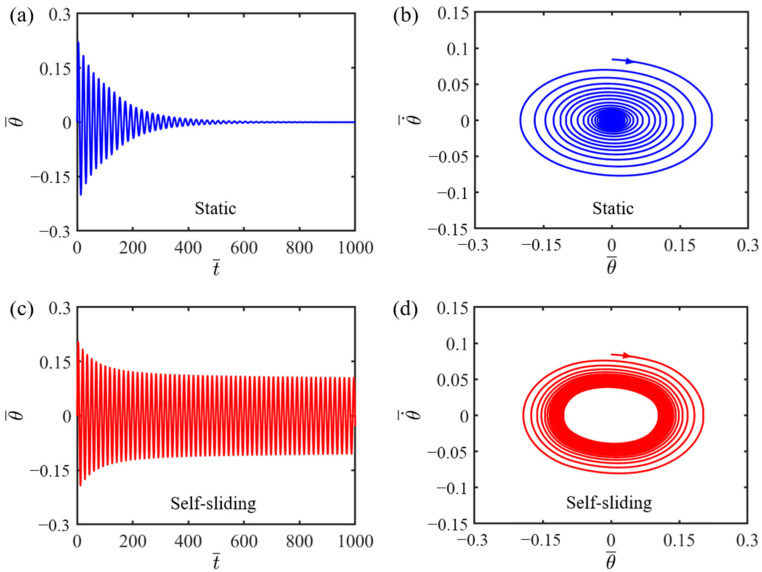
Two typical dynamic modes of the self-sliding structure: static state and self-sliding state. (**a**) Chronological graph of the angular displacement when $\bar{I}=0.15$; (**b**) phase space plot when $\bar{I}=0.15$; (**c**) chronological graph of the angular displacement when $\bar{I}=0.6$; (**d**) the limit cycle in phase space when $\bar{I}=0.6$. If the other dimensionless variables are the same, two distinct dynamic states can be attained under different light intensities.

By numerically solving Equation (11), we can acquire the time–history curve of sliding as well as the phase trajectory diagram for the LCE fiber–sliding mass system, which are depicted in Figure 2. The results show the presence of two clearly distinguishable motion states, namely, the static state and self-sliding state, under different light intensities: $\bar{I}=0.15$ and $\bar{I}=0.6$. During the numerical computation, we set the other dimensionless variables of the sliding mass system, including $C_{0}=0.45$, $\bar{K}=2.7$, $\bar{v_{0}}=0.7$, $\bar{\beta_{1}}=0.015$, $\bar{\beta_{2}}=0.003$, $\theta_{0}=0.09$. For $\bar{I}=0.15$, the system initially slides left and right but eventually comes to a stop due to the damping force, reaching a static state as depicted in Figure 2a. Corresponding to Figure 2a, the phase trajectory in Figure 2b stabilizes at a stationary point. In contrast, as illustrated in Figure 2c,d, where $\bar{I}=0.6$, the sliding amplitude of the LCE fiber–sliding mass system progressively decreases until it reaches a steady value, which is termed the self-sliding mode. Similarly, in the phase trajectory diagram, the amplitude of the sliding mass gradually becomes constant, with its state eventually settling into a limit cycle, signifying a periodic and stable behavior.

### 3.2. Mechanism of Self-Sliding

**Figure 3:**
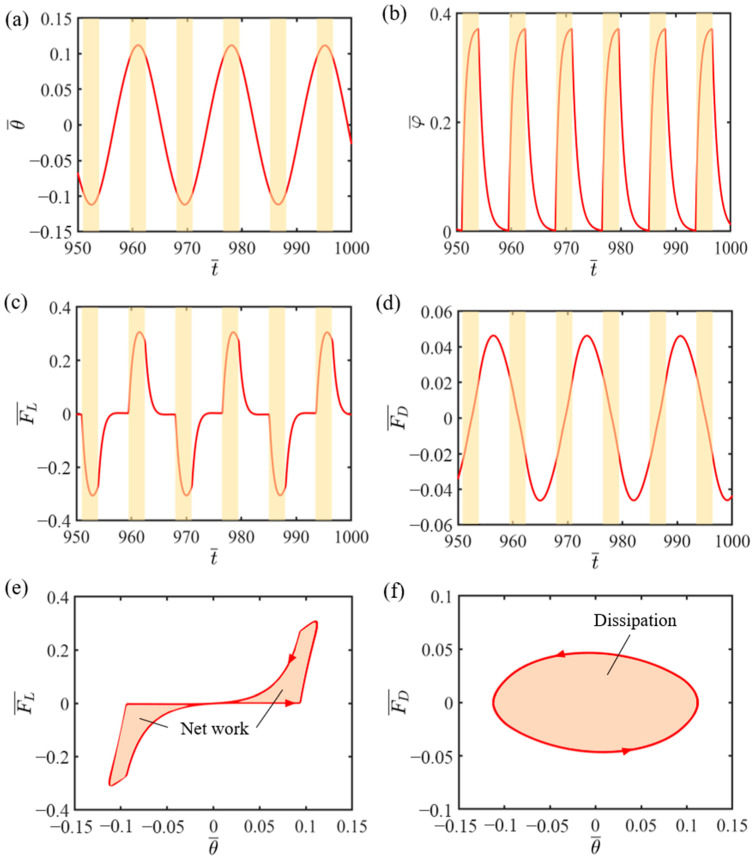
Self-sliding mechanism of the LCE-fiber-based mass system. (**a**) Time–history curve of the angular displacement of the mass. (**b**) Time–history curve of the number fraction of the LCE fiber. (**c**) Variation in the tension of the LCE fiber with time. (**d**) Variation in the damping force with time. (**e**) Dependence of the tension of the LCE fiber on the angular displacement. (**f**) Dependence of the damping force on the angular displacement. Stable self-sliding is maintained in the system due to the light-actuated elastic force, compensating for damping dissipation.

This section is specifically intended to elaborate the self-sliding mechanism, focusing on the inherent energy-balancing mechanism in the system. To aid in a deeper understanding of this complex process, through the plotting of relationship curves, we depict the correlations among several vital variables that are involved in the self-sliding process, as depicted in Figure 3. Herein, the dimensionless variables of the system are selected as $\bar{I}=0.6$, $C_{0}=0.45$, $\bar{K}=2.7$, $\bar{v_{0}}=0.7$, $\bar{\beta_{1}}=0.015$, $\bar{\beta_{2}}=0.003$, and $\theta_{0}=0.09$. Figure 3a depicts the change in the angular displacement of the LCE fiber–sliding mass system over time. The yellow highlighted area denotes the illumination region where the LCE fiber is illuminated. It is apparent that the LCE fiber–sliding mass system maintains a steady amplitude and cycle, with the sliding mass moving to and fro within the illuminated regions on the right and left sides. Figure 3b illustrates how the number fraction in the LCE fiber varies with time due to light exposure. When the angular displacement of the sliding mass is more than the angle of non-illuminated zone $\theta_{0}$, the LCE fiber enters the illuminated regions, and the LCE fiber’s number fraction progressively goes up, approaching a specific maximum. When the sliding mass moves from the illuminated regions into the non-illuminated regions, the LCE fiber’s number fraction sharply decreases to zero. With the system consistently traversing in and out of the illuminated regions, the LCE fiber’s number fraction experiences recurring variations.

Figure 3c demonstrates the time-dependent tension variations in LCE fiber. The tension changes due to the cyclical self-sliding of the system, showing a periodic trend. As the LCE fiber enters the illuminated regions, the tension in the LCE fiber increases as a consequence of the light-actuated shrinkage. However, when the system departs from the illuminated regions, the tension decreases as the light-actuated contraction reverses, as clearly shown in Figure 3c. As is evident from Figure 3d, the damping force displays a periodic variation over time, similar to the tension variations observed in the LCE fiber.

From Figure 3e, it is evident that the hysteresis loop formed by the tension of the LCE fiber illustrates the net work carried out the tension in one complete sliding cycle, which is numerically evaluated to be 0.085. Similarly, Figure 3f reveals the linkage of the damping force with the angular displacement, with the enclosed hysteresis curve signifying the amount of work carried out by the damping force in a full sliding cycle, representing the system’s damping dissipation. Calculations reveal that the area encompassed by the hysteresis loop in Figure 3f also equals 0.085, indicating that the energy dissipated due to the damping force during self-sliding is balanced by the work generated by the tensile force of the LCE fiber. Consequently, the self-sliding of the LCE fiber–sliding mass system remains sustainable.

**Figure 4:**
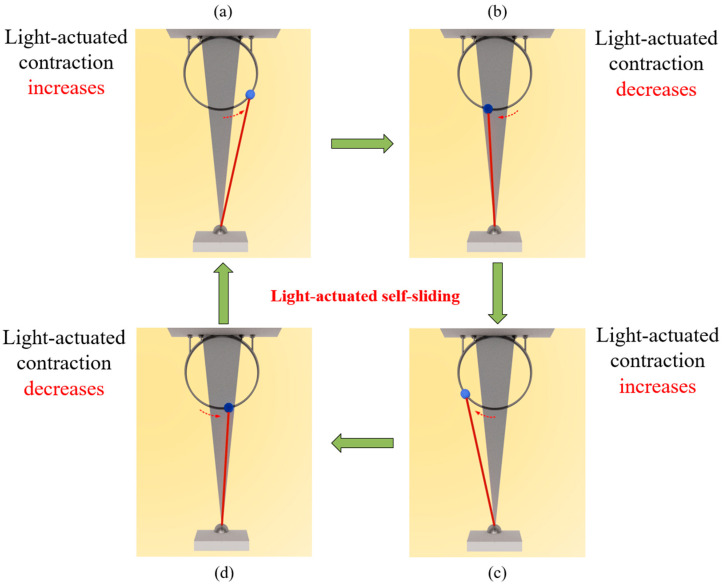
Snapshots of the self-sliding motion in a cycle. (**a**) Move into right illuminated region; (**b**)Sliding in right illuminated region; (**c**) Move into the left illuminated region; (**d**). Sliding in left illuminated region. Under constant light exposure, the periodic changes in contraction actuated by light result in a continual and recurring self-sliding behavior of the system.

Additionally, Figure 4 displays several defining snapshots representing the self-sliding motion of the LCE-fiber-based mass system during a complete sliding cycle under constant light exposure. As the LCE-fiber-based mass system moves from a non-illuminated region to an illuminated region, the increase in the number fraction *φ*(*t*) of the LCE fiber results in a corresponding increase in the contraction strain, reaching a maximum value, as shown in Figure 4a,c. During this process, the system decelerates due to the combined effects of damping forces and the negative work performed via the tension of the LCE fiber, ultimately reducing the velocity to zero. Conversely, when the sliding mass moves from the illuminated regions into the non-illuminated regions, the sharp decrease in the LCE fiber’s number fraction leads to a gradual reduction in the contraction strain, as depicted in Figure 4b,d. Despite this decrease in contraction strain, the tension of the LCE fiber has a positive effect, enabling the system to re-enter the illuminated region and absorb light energy to compensate for the negative effects of damping. This cyclic process allows for continuous self-sliding motion. The variation in the contraction strain of the LCE fiber, as described in Figure 4, aligns with the theoretical framework outlined in the study and specifically with Equation (7).

## 4. Parameter Study

When dynamically modeling the self-sliding behavior of the LCE fiber–sliding mass system, we consider six dimensionless system variables, namely, $\bar{I}$, $C_{0}$, $\bar{K}$, $\bar{v_{0}}$, $\bar{\beta_{1}}$, $\bar{\beta_{2}}$, and $\theta_{0}$. In this section, we undertake a quantitative analysis to examine the impact of these variables on the self-sliding characteristics of the LCE fiber–sliding mass system, particularly highlighting their influence on the amplitude and frequency. Specifically, the dimensionless amplitude and frequency of self-sliding are denoted as A and F, respectively.

### 4.1. Influence of the Light Intensity

**Figure 5:**
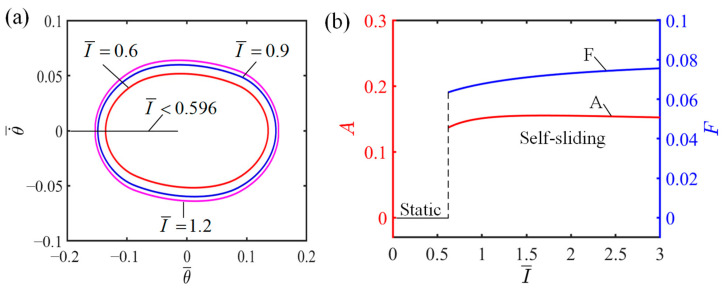
Influence of light intensity on the self-sliding system. (**a**) Limit cycles with $\bar{I}=0.6$, $\bar{I}=0.9$, and $\bar{I}=1.2$. (**b**) Changes in amplitude and frequency with varying light intensities.

Figure 5 demonstrates how light intensity influences the self-sliding, considering specific values of the additional dimensionless variables where $C_{0}=0.45$, $\bar{K}=2.7$, $\bar{v_{0}}=0.7$, $\bar{\beta_{1}}=0.015$, $\bar{\beta_{2}}=0.003$, and $\theta_{0}=0.09$. The corresponding limit cycles of self-sliding for $\bar{I}$ values 0.6, 0.9, and 1.2 are presented in Figure 5a. The amplitude of self-sliding is expressed through the horizontally measured width of the limit cycle, with the vertical height representing the angular velocity of the self-sliding. It can be seen from Figure 5a that the critical light intensity separating the static state from self-sliding state is $\bar{I}=0.596$. At light intensities lower than 0.596, the LCE fiber lacks the necessary light energy absorption to overcome the damping dissipation, resulting in a transition to a static state due to an inability to sustain motion. In contrast, light intensities exceeding 0.596 allow the LCE fiber to absorb sufficient energy to overcome damping dissipation, thereby sustaining continuous and steady self-sliding, which characterizes the self-sliding state. The relationship between light intensity and its influence on amplitude and frequency is shown in Figure 5b. It can be seen from Figure 5b that the amplitude and frequency exhibit a direct correlation as the light intensity rises. The reason for this is stronger light intensities facilitate the LCE fiber’s ability to obtain a greater amount of energy and convert it into kinetic energy, enabling the system to achieve a higher amplitude. The findings indicate that increasing the light intensity plays an important role in enhancing the efficiency of energy utilization within the LCE fiber–sliding mass system.

### 4.2. Influence of the Contraction Coefficient of LCE

**Figure 6:**
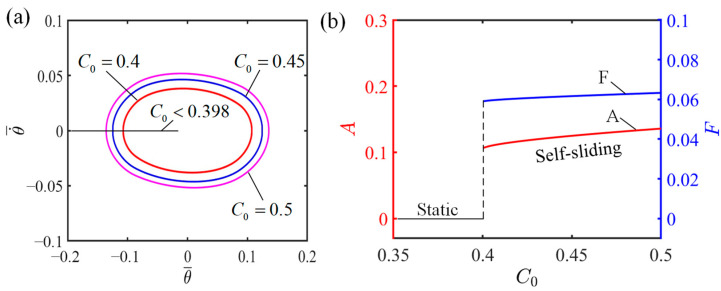
Influence of contraction coefficient on the self-sliding system. (**a**) Limit cycles with $C_{0}=0.4$, $C_{0}=0.45,$ and $C_{0}=0.5$. (**b**) Changes in amplitude and frequency with varying contraction coefficients.

Figure 6 demonstrates how the contraction coefficient influences the self-sliding considering specific values of the additional dimensionless variables, where $\bar{I}=0.6$, $\bar{K}=2.7$, $\bar{v_{0}}=0.7$, $\bar{\beta_{1}}=0.015$, $\bar{\beta_{2}}=0.003$, and $\theta_{0}=0.09$. The corresponding limit cycles of self-sliding for $C_{0}$ values 0.4, 0.45, and 0.5 are presented in Figure 6a. As observed in Figure 6a, the limit cycle associated with a higher contraction coefficient completely envelopes the one with a lower coefficient, showing that a decrease in the contraction coefficient weakens the LCE fiber–sliding mass system’s ability to absorb light, leading to a decreased amplitude and kinetic energy. Figure 6b illustrates how the amplitude and frequency of self-sliding are influenced by the contraction coefficient. As observed in Figure 6b, a distinct threshold value exists for the contraction coefficient, which is mathematically determined to be 0.398, and serves as the tipping point for inducing self-sliding. Below a contraction coefficient of 0.398, the sliding mass maintains a stationary condition. However, once the contraction coefficient rises above 0.398, the system enters a self-sliding state. The finding suggests that the efficient conversion of light energy to mechanical energy can be improved by increasing the contraction coefficient of the LCE fiber.

### 4.3. Influence of the Elastic Coefficient of LCE

**Figure 7:**
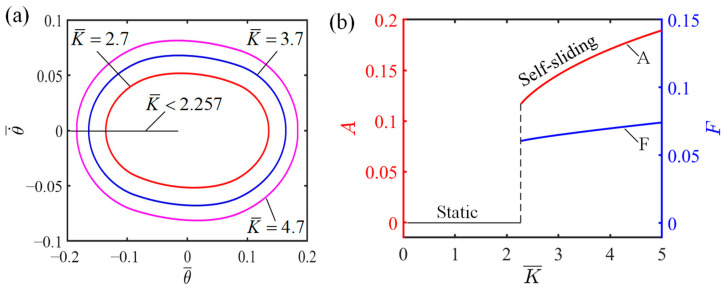
Influence of elastic coefficient on the self-sliding system. (**a**) Limit cycles with $\bar{K}=2.7$, $\bar{K}=3.7$, and $\bar{K}=4.7$ (**b**) Changes in amplitude and frequency with varying elastic coefficients.

Figure 7 demonstrates how the elastic coefficient influences the self-sliding, considering the specific values of the additional dimensionless variables, where $\bar{I}=0.6$, $C_{0}=0.45$, $\bar{v_{0}}=0.7$, $\bar{\beta_{1}}=0.015$, $\bar{\beta_{2}}=0.003$, and $\theta_{0}=0.09$. For $\bar{K}=2.7$, $\bar{K}=3.7$, and $\bar{K}=4.7$, the corresponding limit cycles of self-sliding are presented in Figure 7a. From Figure 7a, it is evident that $\bar{K}=2.257$ represents the threshold value of the elastic coefficient that determines whether the system remains in static mode or self-sliding mode. Under steady illumination, if the elastic coefficient is smaller than 2.257, the LCE fiber is unable to absorb adequate light energy. Consequently, the system lacks sufficient energy to counteract damping dissipation and eventually settles into a static state. Conversely, when the elastic coefficient is more than 2.257, the LCE fiber has the ability to absorb an ample amount of energy, counteracting the system’s damping dissipation and thereby sustaining self-sliding. As depicted in Figure 7b, the elastic coefficient plays a vital role in determining the magnitude and periodicity of the self-sliding. As the elastic coefficient increases, there is a corresponding increase in the magnitude and periodicity of self-sliding. This is because a greater elastic coefficient generates a stronger elastic force from the LCE fiber. As a result, the system gains more elastic potential energy that can be converted into kinetic energy, thus leading to a higher amplitude of self-sliding. Therefore, selecting the appropriate elastic coefficient is essential in achieving superior performance when designing an LCE-based tension system.

### 4.4. Influence of the Initial Tangential Velocity

**Figure 8:**
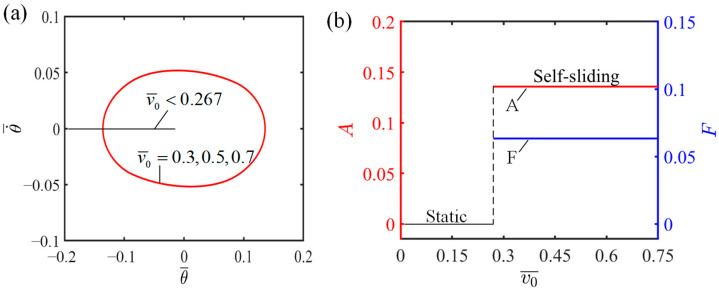
Influence of initial tangential velocity on the self-sliding system. (**a**) Limit cycles with $\bar{v_{0}}=0.3$, $\bar{v_{0}}=0.5,$ and $\bar{v_{0}}=0.7$. (**b**) Changes in amplitude and frequency with varying initial tangential velocities.

Figure 8 demonstrates how initial tangential velocity influences the self-sliding, considering specific values of the additional dimensionless variables where $\bar{I}=0.6$, $C_{0}=0.45$, $\bar{K}=2.7$, $\bar{\beta_{1}}=0.015$, $\bar{\beta_{2}}=0.003$, and $\theta_{0}=0.09$. The numerical simulations reveal that initiating self-sliding in the LCE fiber–sliding mass system is possible with initial tangential velocities of $\bar{v_{0}}=0.3$, $\bar{v_{0}}=0.5,$ and $\bar{v_{0}}=0.7$. The limit cycles resulting from these velocities are presented in Figure 8a and it is worth mentioning that all three limit cycles overlap.

Figure 8b represents the dependency of the characteristics of self-sliding on the initial tangential velocity, namely its amplitude and frequency. Based on Figure 8b, it is evident that the system enters a static state when the initial tangential velocity is below 0.267. This occurs because the insufficient initial tangential velocity prevents the LCE fiber from accessing the illumination zone, thereby hindering the absorption of the light energy necessary to maintain dynamic motion, ultimately resulting in a static state. When the initial tangential velocity exceeds 0.267, given $\bar{v_{0}}=0.3$, $\bar{v_{0}}=0.5$, and $\bar{v_{0}}=0.7$, the system enters a self-sliding state, during which the ultimate amplitude and frequency are unaffected by the variable initial tangential velocities. The reason for this is that the amplitude of self-sliding is determined by the energy conversion process between damping dissipation and the net work carried out by the LCE fiber, which form the system’s internal properties. Since the initial tangential velocity has no impact on the system’s energy conversion mechanism, the amplitude remains unaffected.

### 4.5. Influence of the Damping Coefficients

**Figure 9:**
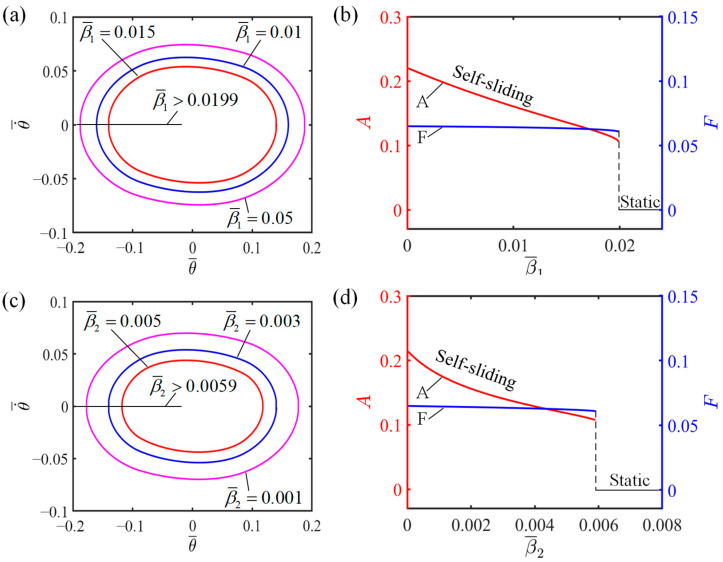
Influence of damping coefficients on the self-sliding system. (**a**) Limit cycles with $\bar{\beta_{1}}=0.005$, $\bar{\beta_{1}}=0.01$, and $\bar{\beta_{1}}=0.015$. (**c**) Limit cycles with $\bar{\beta_{2}}=0.001$, $\bar{\beta_{2}}=0.003$, and $\bar{\beta_{2}}=0.005$. (**b**,**d**) Changes in amplitude and frequency with varying damping coefficients.

Figure 9 demonstrates how damping coefficients influence the self-sliding, considering specific values of the additional dimensionless variables where $\bar{I}=0.6$, $C_{0}=0.45$, $\bar{K}=2.7$, $\bar{v_{0}}=0.7$, and $\theta_{0}=0.09$.

Figure 9a,c further illustrate that as the damping coefficient increases, the limit cycle decreases in size. When the damping coefficient surpasses a certain critical value, the system transitions from a self-sliding state to a static state. As the damping coefficient rises, a greater amount of energy becomes necessary to overcome the energy dissipation that occurs due to the damping forces, thus marking this transitional change. The energy harvested by the LCE in the illuminated zone becomes insufficient to overcome the damping forces, ultimately leading the system to settle into a static state. In practical system design, it is imperative to devise strategies that minimize energy losses due to resistance, enabling the energy generated by the LCE fiber in the illuminated zone to be more efficiently utilized for the system’s intrinsic self-sliding motion. From Figure 9b,d, it is evident that whether it is $\bar{\beta_{1}}$ or $\bar{\beta_{2}}$, a smaller damping coefficient results in a larger amplitude of self-sliding, and vice versa. The amplitude is significantly affected by the damping coefficient, whereas the impact on the frequency of self-sliding is less significant.

### 4.6. Influence of the Angle of Non-Illuminated Zone

**Figure 10:**
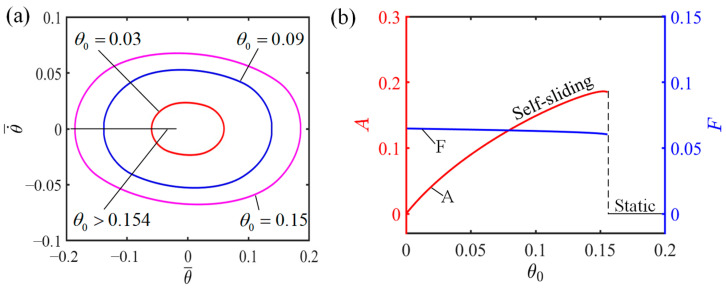
Influence of the angle of the non-illuminated zone on the self-sliding system. (**a**) Limit cycles with $\theta_{0}=0.03$, $\theta_{0}=0.09$, and $\theta_{0}=0.15$. (**b**) Changes in amplitude and frequency with varying angles of non-illuminated zone.

Figure 10 demonstrates how non-illuminated zone angle influences the self-sliding, considering specific values of the additional dimensionless variables where $\bar{I}=0.6$, $C_{0}=0.45$, $\bar{K}=2.7$, $\bar{v_{0}}=0.7$, $\bar{\beta_{1}}=0.015$, and $\bar{\beta_{2}}=0.003$. For $\theta_{0}=0.03$, $\theta_{0}=0.09$, and $\theta_{0}=0.15$, the corresponding limit cycles of self-sliding are presented in Figure 10a. It can be seen from Figure 10a that the critical angle of the non-illuminated zone separating the static state from the self-sliding state is $\theta_{0}=0.154$. Once the angle of the non-illuminated zone exceeds 0.154, the LCE fiber is incapable of entering the illuminated zone to gather sufficient light energy to overcome the damping-induced energy loss. Consequently, due to the exhaustion of the kinetic energy, the system inevitably transitions into a static state. In contrast, when the angle of the non-illuminated zone is less than 0.154, the LCE fiber is able to reach the illuminated zone to absorb sufficient energy to overcome damping dissipation, thereby sustaining continuous and steady self-sliding, which characterizes the self-sliding state. The relationship between the angle of the non-illuminated zone and its influence on amplitude and frequency is shown in Figure 10b. It can be seen from Figure 10b that the amplitude exhibits a direct correlation with the increase in the angle of the non-illuminated zone. The explanation lies in the fact that, with a small angular non-illumination, the LCE fiber promptly enters the illuminated zone, causing its increasing tension to restrict the sliding mass from continuing its forward movement and thereby limiting the amplitude of self-sliding. In contrast, a larger angular non-illumination allows for greater angular displacement of the LCE fiber–sliding mass system before it reaches the illuminated zone, subsequently enhancing the total amplitude of self-sliding.

## 5. Conclusions

Existing self-vibrating systems, despite offering adaptability, efficiency, and sustainability, are complex, hard to produce, and challenging to control. This highlights the urgency of developing more advanced and practical self-oscillation structures. In the study, we propose a light-actuated LCE fiber–sliding mass system consisting of a light-actuated LCE fiber, a sliding mass, and a rigid circular track, which can sustain periodic and continuous sliding under constant light exposure. Derived from the available dynamic behavior model of LCEs, and the theorem of angular momentum and vibration theory, the equations for dynamic control of the sliding mass enabled by the light-actuated LCE fiber are deduced. Using the standard fourth-stage Runge–Kutta method and MATLAB R2021a software, the numerical calculation of the dynamic control equations is acquired. According to the results, two motion states of the self-sliding mass system are explained in detail, which are categorized as the static state and self-sliding state. In particular, the self-sliding process, along with its energy balancing system, is clarified. Herein, the energy drawn from a constant external source serves to balance out the dissipation caused by system damping, preserving its dynamic equilibrium.

Moreover, the influences of light intensity, contraction coefficient, elastic coefficient, initial tangential velocity, linear and quadratic damping coefficients, and angle of the non-illuminated zone are quantitatively analyzed. The numerical calculation results reveal an increase in light intensity, contraction coefficient, elastic coefficient, and angle of the non-illuminated zone, which leads to an augmentation in both the amplitude and frequency of the system. Specifically, the elastic coefficient and the angle of the non-illuminated zone exert a profound influence on the amplitude, with each π/180 increment in amplitude leading to a 0.25% increase in the elastic coefficient and a 1.63% expansion in the angle of the non-illuminated zone. Additionally, light intensity plays a crucial role, contributing to a 20.88% augmentation for every increment of π/180 in the amplitude. In contrast, while an increase in the damping coefficient leads to a notable reduction in both the amplitude and frequency, the initial tangential velocity has no discernible effect on either the amplitude or the frequency of the system.

While the simplicity, controllability, and rapid response to the light of the proposed LCE fiber–sliding mass system render it a promising candidate for widespread adoption, it is important to acknowledge the limitations of the small deformation assumption, the simplified damping consideration, and the neglect of viscoelastic effects in LCE fibers. To further enhance the system’s potential for broad applications, future research will aim to incorporate viscoelasticity into the model and investigate the system’s performance on non-circular curved tracks.

## Figures and Tables

**Table 1:** Material properties and geometric parameters.

Parameter	Definition	Value	Unit
I	light intensity	0~80	kW/m^2^
$C_{0}$	contraction coefficient of LCE fiber	0~0.5	/
K	elastic coefficient of LCE fiber	20~40	N/m
$T_{0}$	*Cis* to *trans* thermal relaxation time	0.02~0.45	s
$\eta_{0}$	light absorption constant	0.002	m^2^/(s·W)
m	mass of sliding mass	0~0.02	kg
$\beta_{1}$	linear damping coefficient	0~0.3	kg/s
$\beta_{2}$	quadratic damping coefficient	0~0.15	kg/m
$v_{0}$	initial tangential velocity	0~2.5	m/s
$\theta_{0}$	angle of non-illuminated zone	0~0.5	rad
r	radius of circular track	0.01~0.15	m
$L_{0}$	original length of LCE fiber	0.1~0.5	m

**Table 2:** Dimensionless parameters.

Parameter	$\bar{I}$	$C_{0}$	$\bar{K}$	$\bar{v_{0}}$	$\bar{\beta_{1}}$	$\bar{\beta_{2}}$	$\theta_{0}$
Value	0~5	0~0.5	0~10	0~1	0~0.2	0~0.1	0~0.5

## Data Availability

The original contributions presented in the study are included in the article/Supplementary Material, further inquiries can be directed to the corresponding authors.

## Supplementary Materials

The following supporting information can be downloaded at: https://www.mdpi.com/article/10.3390/polym16121696/s1, Video S1: The process of the self-sliding motion of the system in a cycle.
